# CLRM-02. TRIAL IN PROGRESS: A PROSPECTIVE, MULTICENTER PHASE 2B STUDY TO ESTABLISH IMAGE INTERPRETATION CRITERIA FOR ^18^F-FLUCICLOVINE PET IN DETECTING RECURRENT BRAIN METASTASES AFTER RADIATION THERAPY (PURSUE)

**DOI:** 10.1093/noajnl/vdab112.001

**Published:** 2021-09-21

**Authors:** Rupesh Kotecha, Alain Chaglassian, Nancy Tainer, Eugene J Teoh

**Affiliations:** 1 Department of Radiation Oncology, Miami Cancer Institute, Baptist Health South Florida, Miami, FL, USA; 2 Blue Earth Diagnostics Inc., Burlington, MA, USA; 3 Blue Earth Diagnostics Ltd, Oxford, United Kingdom

## Abstract

**BACKGROUND:**

Brain metastases represent the most common intracranial tumor in adults, occurring in 10-40% of cancer patients. Most patients undergo multimodal treatment approaches and post-treatment follow-up with conventional MRI (CE-T1-weighted and FLAIR/T2-weighted) of the brain is performed to monitor for disease recurrence. However, owing to the similar appearance of treatment-related changes like radiation necrosis with that of true recurrence, conventional MRI alone suffers from low specificity. Given the high mortality of patients with brain metastases and the considerable treatment-associated morbidity, a need remains for an imaging modality that accurately differentiates recurrence from treatment-related changes. Accurate imaging is key to preventing unnecessary surgery or changes in effective therapy in patients mistaken for disease progression as well as prevent continuation of ineffective therapy if radiation necrosis is incorrectly diagnosed. To this end, ^18^F-fluciclovine is a synthetic amino acid-based PET imaging agent that has potential to evaluate primary and metastatic brain cancers owing to its low normal background uptake in the brain and increased uptake in brain tumors.

**METHODS:**

NCT04410367 is a prospective, open-label, single-arm, single-dose (185 MBq ± 20%) study with a primary objective to establish visual image interpretation criteria for ^18^F-fluciclovine PET studies of recurrent brain metastases. Forty subjects with solid tumor brain metastases who have undergone radiation therapy will be enrolled across ~8 US sites if they have a reference lesion considered equivocal on MRI for recurrent disease and are planned for craniotomy. Subjects will undergo ^18^F-fluciclovine PET <42 days after the MRI and 1–21 days before planned craniotomy. Outcome measures comprise the diagnostic performance of ^18^F-fluciclovine PET at different thresholds of ^18^F-fluciclovine uptake compared with histopathology, subject- and lesion-level diagnostic performance based on established image interpretation criteria, and safety evaluations. Enrolment began in August 2020 and the trial is open at the time of submission.

